# A gD&gC-substituted pseudorabies virus vaccine strain provides complete clinical protection and is helpful to prevent virus shedding against challenge by a Chinese pseudorabies variant

**DOI:** 10.1186/s12917-018-1766-8

**Published:** 2019-01-03

**Authors:** Chuanjian Zhang, Yamei Liu, Saisai Chen, Yongfeng Qiao, Mingpeng Guo, Yating Zheng, Mengwei Xu, Zhisheng Wang, Jibo Hou, Jichun Wang

**Affiliations:** 1 Institute of Veterinary Immunology and Engineering, National Research Center of Engineering and Technology for Veterinary Biologicals, Jiangsu Key Laboratory for Food Quality and Safety-State Key Laboratory Cultivation Base of the Ministry of Science and Technology, Jiangsu Academy of Agricultural Sciences, Nanjing, 210014 Jiangsu China; 2 Jiangsu Co-innovation Center for Prevention and Control of Important Animal Infectious Diseases and Zoonoses, Yangzhou, 225009 Jiangsu China

**Keywords:** Bacterial artificial chromosome, gD&gC substitution, Immunogenicity, Pseudorabies virus, Safety

## Abstract

**Background:**

Since 2011, pseudorabies caused by a variant PRV has re-emerged in many Chinese Bartha-K61-vaccinated pig farms. An efficacious vaccine is necessary to control this disease. We described the construction of a gD&gC-substituted pseudorabies virus (PRV B-gD&gC^S^) from the Bartha-K61 (as backbone) and AH02LA strain (as template for gD and gC genes) through bacterial artificial chromosome (BAC) technology using homologous recombination. The growth kinetics of PRV B-gD&gC^S^ was compared with Bartha-K61. Its safety was evaluated in 28-day-old piglets. Protection efficacy was tested in piglets by lethal challenge with AH02LA at 7 days post vaccination, including body temperature, clinical symptoms, virus shedding, mortality rate, and lung lesions.

**Results:**

The results showed that a BAC clone of Bartha-K61 and a B-gD&gC^S^ clone were successfully generated. The growth kinetics of PRV B-gD&gC^S^ strain on ST (Swine testicular) cells was similar to that of the Bartha-K61 strain. No piglets inoculated intramuscularly with PRV B-gD&gC^S^ strain exhibited any clinical symptoms or virus shedding. After AH02LA challenge, all piglets in PRV B-gD&gC^S^ and Bartha-K61 groups (*n* = 5 each) survived without exhibiting any clinical symptoms and high body temperature. More importantly, PRV B-gD&gC^S^ strain completely prevented virus shedding in 2 piglets and reduced virus shedding post challenge in the other 3 piglets as compared with Bartha-K61 group.

**Conclusions:**

Our results suggest that PRV B-gD&gC^S^ strain is a promising vaccine candidate for the effective control of current severe epidemic pseudorabies in China.

## Background

Pseudorabies is a highly contagious and economically significant porcine infectious disease in many countries [1–4]. The pathogen pseudorabies virus (PRV) is an alpha-herpesvirus belonging to the family Herpesviridae [5]. Swine are the major host and the only natural reservoir of the virus [6]. PRV infection may cause fatal encephalitis and high mortality in piglets, respiratory illness and growth retardation in growing pigs, as well as abortions and stillbirths in sows [7]. Pseudorabies has been eradicated in the domestic swine of the United States, some European countries and New Zealand via immunization of gE-deleted vaccines (especially Bartha-k61 vaccine) [6, 8, 9]. Nevertheless, PRV is still circulating and remains a serious threat to the domestic swine industry in many regions, especially in several developing countries. In China, vaccination with the standard attenuated live vaccine strain Bartha-K61 imported from Hungary in the 1970 has been the primary measure for the prevention and control of pseudorabies, and the disease was well controlled until 2011 when large-scale outbreaks of pseudorabies caused by new emerging PRV variants took place in many pig herds [10]. Previous studies have shown that pigs with Bartha K61 vaccine had mild or no clinical symptoms post PRV virulent strain challenge, but developed high viral shedding titers [11, 12]. The eradication of PRV has been an ultimate aim of many farms to control this disease. No or significant reduction in virus shedding post virulent strain infection is crucial to achieve this goal. It is therefore urgent to develop a more efficient vaccine to control the virus shedding and to ultimately eradicate the disease.

The PRV genome is a 143-kb-long linear double-stranded DNA encoding for approximately 70 genes, including 11 different glycoproteins (gB, gC, gD, gE, gG, gH, gI, gK gL, gM, and gN) [5, 13]. gB, gC and gD have been suggested as important antigens that induce protective immunity against PRV infection [14, 15]. Vaccination of recombinant heterologous vectors expressing gB, gC and gD induces either antibody- or cell-mediated immunity and elicits protection in mice or pigs [15–18]. We have previously isolated a highly virulent PRV strain, named AH02LA [19], which has remarkable amino acid mutations in glycoproteins including gB, gC and gD (95–98% homology) when compared with those in classical PRV strains (Bartha-K61 and Kaplan strains) [20]. The other recent virulent PRV isolates from various regions in China also harbor substantial sequence mutations in glycoproteins compared with the classic strains [21, 22]. More importantly, all these virulent variants share a high sequence homogeneity of 99–100%, and are clustered into an independent branch in the phylogenetic tree that is antigenically distant from the classical PRVs [21, 23]. This genetic divergence in glycoproteins among virulent PRV variants and the classical PRVs directly led to the reduced protection efficiency of Bartha-K61 vaccination against the severe PRV epidemic. A previous study showed that inactivated Bartha-K61 strain with gB-substituted by PRV variant JS-2012 genes increased protective efficacy to 80% against variant JS-2012 strain compared with Bartha-K61 strain (40%) in mice [24]. Therefore, we hypothesize that a recombinant Bartha-K61 strain with gD&gC-substituted by AH02LA genes would have both high safety and excellent immunogenicity for piglets. Meanwhile, considering the high genetic homogeneity among PRV virulent strains, we believe that gD&gC-substituted pseudorabies virus vaccine strain might be a promising vaccine candidate to control the current circulating virulent PRV variants in China although challenge experiment was only conducted with AH02strain in this study.

In this study, we generated a gD&gC-susbtituted PRV mutant from the Bartha-K61 (as backbone) and AH02LA strain (as template for gD and gC genes) through bacterial artificial chromosome (BAC) technology using homologous recombination, and evaluated its growth kinetics as well as safety and immunogenicity for piglets. Our results showed that the gD&gC-substituted pseudorabies virus vaccine strain could not only provide complete clinical protection but also effectively reduce and may even prevent virus shedding against lethal AH02LA challenge, indicating that it might be an efficient vaccine to control this emergence disease in China.

## Results

### Construction of a BAC containing the Bartha-K61 genome

**Fig. 1:**
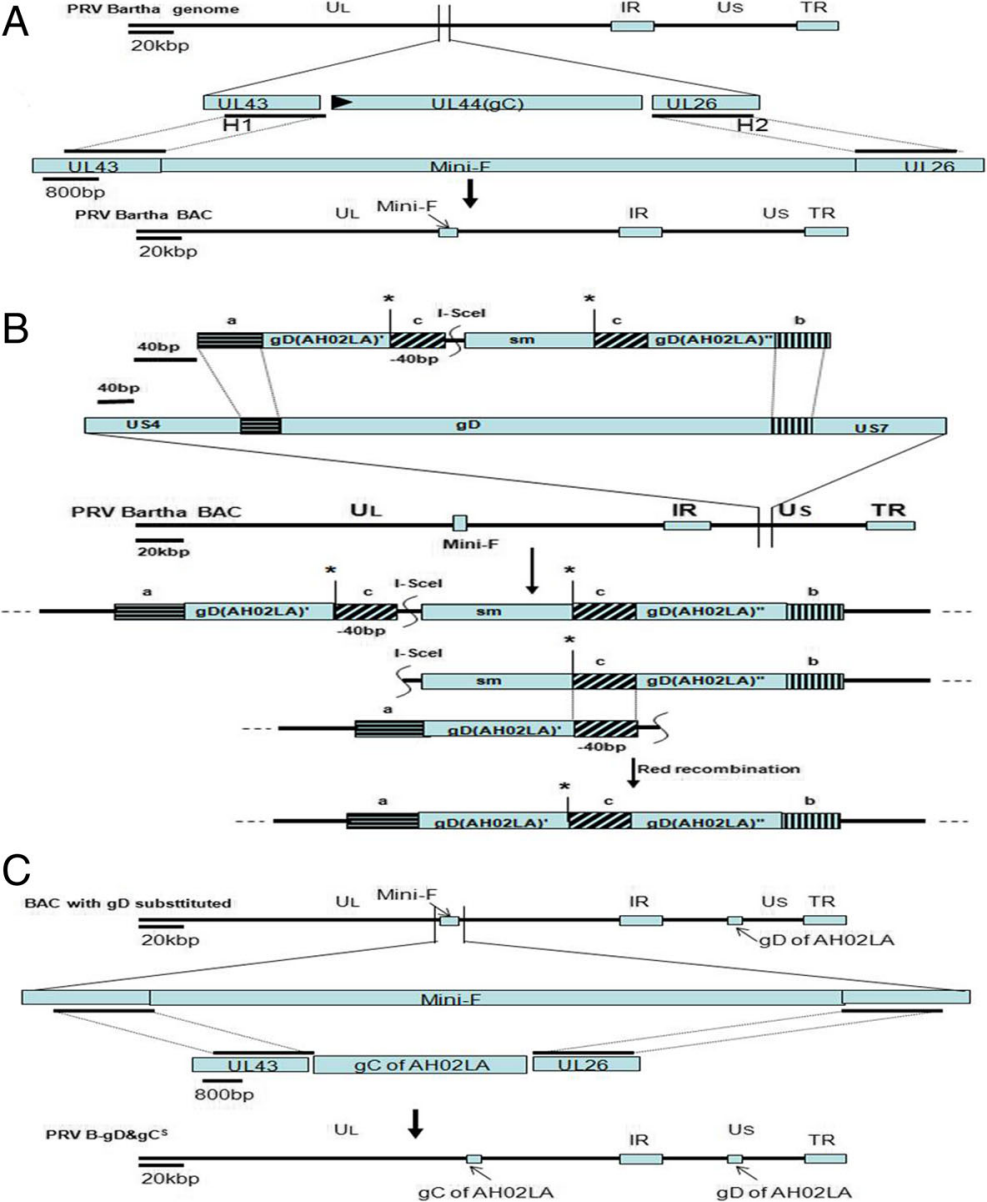
Construction of mini-F recombinant PRV Bartha strain (PRV B-mini-F), gD substituted clone (pB-gD^S^-mini-F) and gC&gD-substituted virus (PRV B-gD&gC^S^) (**a**) Mini-F was inserted in lieu of gC to generate the mini-F recombinant PRV Bartha-K61 strain for BAC through homologous recombination. **b** AH02LA gD-KAN was inserted in lieu of gD in the Bartha genome through the first recombination, the kanamycin gene was deleted in the second step, generating gD substituted clone (pB-gD^S^-mini-F). **c** Another recombination was performed to substitute the mini-F sequence with gC of AH02LA, generating gC&gD substituted virus (PRV B-gD&gC^S^)

**Fig. 2:**
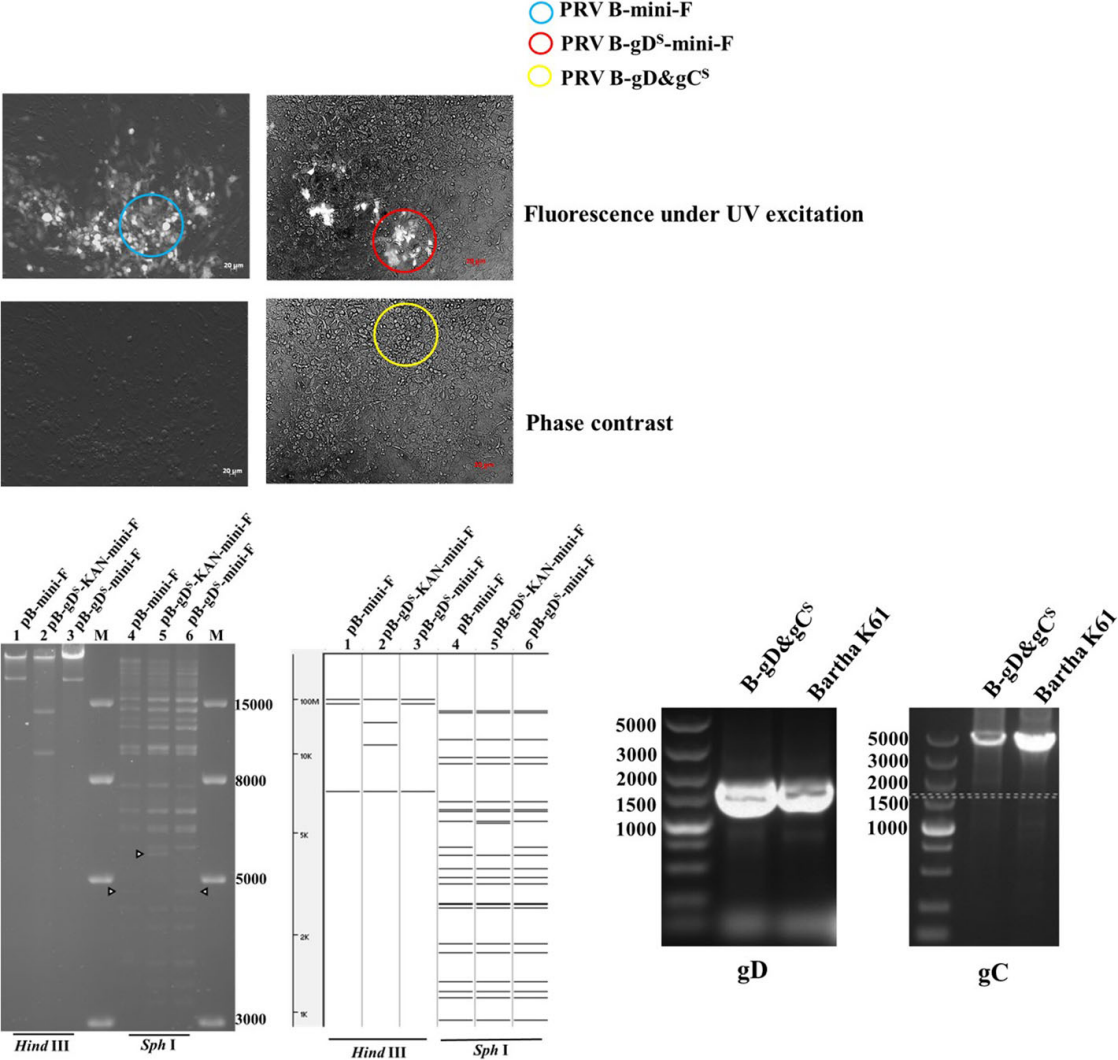
Plaque of PRV B-mini-F, PRV B-gD^S^-mini-F and PRV B-gD&gC^S^, RFLP of pB-mini-F, pB-gD^S^-KAN-mini-F and pB-gD^S^-mini-F, and PCR verification of gC and gD genes replacement. A Images of PRV B-mini-F, PRV B-gD^S^-mini-F and PRV B-gD&gC^S^ plaques under UV excitation and contrast are shown. B DNA from pB-mini-F BAC clone (lanes 1 and 4) and recombinant BACs of pB-gD^S^-KAN-mini-F (lanes 2 and 5) and pB-gD^S^-mini-F (lanes 3 and 6) were prepared by mini-prep and digested with *Hind* III (lanes 1–3) or *Sph* I (lanes 4–6). Digests were separated by 0.8% agarose gel electrophoresis for 15 h under 40 V. Predictions of these digestions were performed using whole genome sequences of Bartha-K61 as a reference (GenBank ID: JF797217.1). C Verification of gC and gD genes replacement by PCR. gD of Bartha-K61 and PRV B-gD&gC^S^ were identified with AH02LA-gD-F/AH02LA-gD-R. gC of Bartha-K61 and PRV B-gD&gC^S^ were identified with SEQ-AH02LA gC F/SEQ-AH02LA gC R

Green plaques of recombinant PRV Bartha-K61 with mini-F that replaced the gC gene were observed under UV light (488 nm) at 48 h after co-transfection of Bartha-K61 DNA and the BAC transfer vector plasmid pHA2-Puc19-H1^B^-H2^B^ (Fig. 2a). After three rounds of plaque purification, a homogeneous population of recombinant PRV with mini-F (PRV B-mini-F) was obtained. B-mini-F DNA was electroporated into *E.coli* DH10B competent cells. The obtained positive clone (pB-mini-F) DNA was transfected into GS1783 competent cells, generating the infectious clone for further gene manipulations through the *En Passant* method (Fig. 1).

### Construction of PRV recombinant BAC (pB-gD^S^-mini-F)

Based on infectious clone, gD of Bartha was replaced with gD of AH02LA containing a kanamycin resistance gene through the first recombination. Through a second recombination, the kanamycin resistance gene was deleted, generating the PRV recombinant BAC (pB-gD^S^-mini-F) (Fig. 1). RFLP analysis of pB-mini-F, pB-gD^S^-KAN-mini-F and pB-gD^S^-mini-F with *Hind* III or *Sph* I corresponded with the predicted pattern with minor differences (Fig. 2b). The replaced gD was confirmed by PCR and sequencing (data not shown).

### Generation of gD&gC-substituted pseudorabies virus (PRV B-gD&gC^S^)

To generate PRV B-gD&gC^S^, co-transfection of DNA of pB-gD^S^-mini-F and H1-H2-gC^A^-T, non-fluorescent plaques were observed under UV light (488 nm) (Fig. 2a). To obtain a homogeneous population, one plaque was isolated after 5 rounds of plaque purification and named PRV B-gD&gC^S^. The replaced gC and gD were confirmed by PCR and sequencing (Fig. 2c).

### Growth kinetics of PRV B-gD&gC^S^

**Fig. 3:**
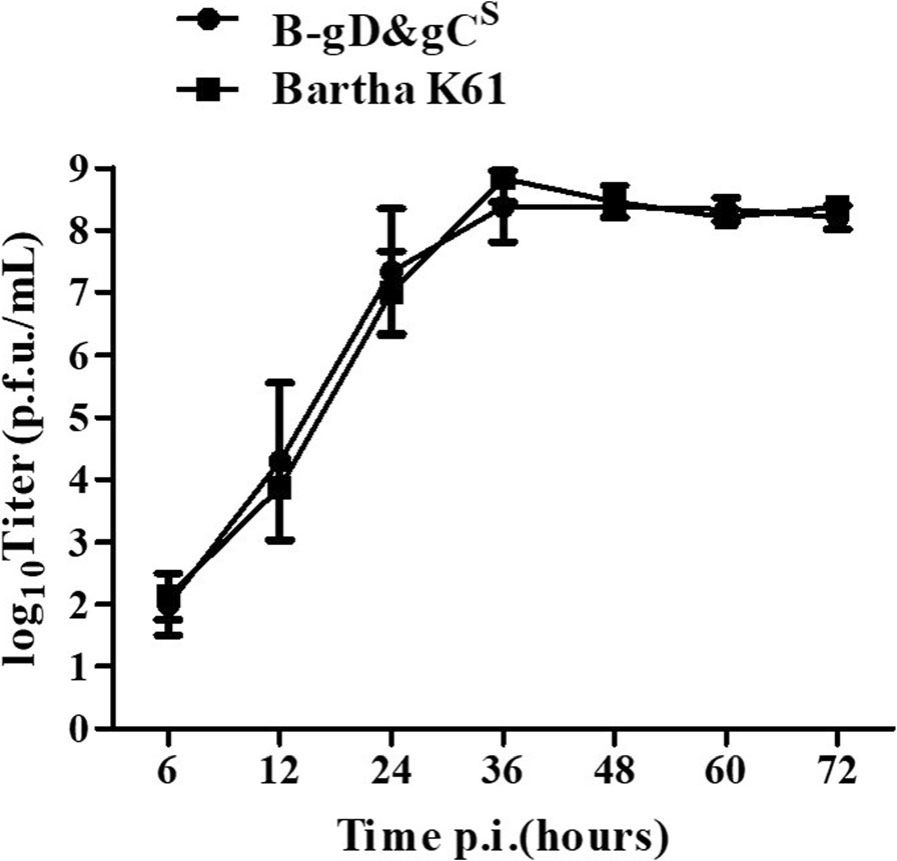
Multi-step growth curves of Bartha-K61 and PRV B-gD&gC^S^ on STs. At 0, 6, 12, 24, 36, 48, 60 and 72 h post infection, virus was titrated on STs with a MOI of 0.01. Data were presented as mean ± SD, and analyzed using Student’s t test by SPSS 16.0 (SPSS Inc., Chicago, IL, USA)

The growth kinetics of the Bartha-K61 and PRV B-gD&gC^S^ viruses on ST cells were shown in Fig. 3. The growth kinetics of PRV B-gD&gC^S^ were similar to those of Bartha-K61. Peak titers for Bartha-K61 and PRV B-gD&gC^S^ were 10^8.83^ and 10^8.38^ TCID_50_/mL respectively (Fig. 3).

### Safety and immunogenicity of PRV B-gD&gC^S^ in piglets

Piglets inoculated intramuscularly with PRV B-gD&gC^S^ remained healthy, without fever, clinical signs and virus shedding during the 1-week observation period (Table 1), suggesting that PRV B-gD&gC^S^ is safe for piglets.

For immunogenicity, after AH02LA challenge, all piglets in challenge control group showed typical clinical syndromes including prolonged high fever, sneezing, coughing, dyspnea and spirits atrophy. No clinical symptom was observed in any piglets in PRV B-gD&gC^S^ or Bartha-K61 group (Table 1). The piglets in challenge control group showed severe lung lesions (Table 1). The piglets in PRV B-gD&gC^S^ and Bartha-K61groups have no lung lesions. Moreover, one piglet died at 6 dpc, one at 7 dpc and one at 8 dpc in challenge control group (Table 1). For gB and gE antibodies, gB specific antibody was detected positive in all piglets of PRV B-gD&gC^S^ and Bartha-K61groups at 7 dpi (Table 1). At 14 dpc, the gB and gE specific antibodies were detected positive in all piglets in PRV B-gD&gC^S^ and Bartha-K61 groups or survived piglets in challenge control group (Table 1). No gE and gB specific antibodies were developed in PBS control group (Table 1). As shown in Table 1, virus shedding was found in all pigs of challenge control group with titers of 10^1.30^~10^8.83^ TCID_50_/g from 3 to 10 dpc. The titers of shed virus in piglets inoculated intramuscularly with Bartha-K61 were 10^1.30^~10^9.82^ TCID_50_/g from 4 to 10 dpc. In the PRV B-gD&gC^S^group, two piglets did not shed viruses, and the titers of shed viruses of the other three pigs ranged from 10^2.49^ to 10^6.19^ TCID_50_/g between 1 and 8 dpc. The levels and duration of neutralizing antibodies and cell-mediated immunity are also important parameters to evaluate the efficacy of PRV vaccine. Therefore, future studies involving the neutralizing antibody and cell-mediated immunity analysis are necessary to better compare performance differences between B-gD&gC^S^ and Bartha-K61 groups. All together, our results suggest that PRV B-gD&gC^S^ is safe for piglets, and provides complete clinical protection against a pseudorabies variant (AH02LA) challenge. More importantly, it greatly reduces and may even prevents virus shedding in piglets challenged with virulent AH02LA strain.

## Discussion

Since 2011, pseudorabies caused by a variant PRV has re-emerged in Bartha-K61-vaccinated pig farms in China [23]. Previous study showed that PRV Bartha K61 vaccine failed to stop virus shedding in pigs against highly virulent PRV strains [25, 26]. The safety of gene deletion mutants from the new variant is far from satisfactory [12]. Therefore, developing a new safe vaccine is necessary to control and eradicate the virulent PRV variant. In this study, we constructed a gD&gC-substituted pseudorabies virus (PRV B-gD&gC^S^) from the Bartha-K61 (as backbone) and AH02LA strain (as template for gC and gD genes). The growth kinetics of PRV B-gD&gC^S^ were similar to that of Bartha-K61. Piglets experiments showed that PRV B-gD&gC^S^ is safe for piglets. Furthermore, it provides complete clinical protection, and greatly reduces and may even prevent virus shedding against a pseudorabies variant (AH02LA) challenge.

Mutants of herpesvirus were generated by the BAC mutagenesis protocol to study the mechanism of the viruses and construct vectored vaccines [27]. In the study, we construct Bartha-K61 BAC with gC replaced by mini-F sequences. Based on Bartha-K61 BAC, gD of Bartha was replaced with gD of AH02LA though *En Passant* protocol. PRV B-gD&gC^S^ was obtained by homologous recombination between pB-gD^A^-mini-F and H1-H2-gC^A^-T. Compared to the parental Bartha-K61, PRV B-gD&gC^S^ showed similar growth properties on ST cells.

Variant PRV infections cause high mortality and clinical symptoms including fever, and respiratory and neurologic symptoms in piglets [28]. In the current study, the piglets of PRV B-gD&gC^S^ groups showed no clinical pseudorabies symptoms post inoculation. After AH02LA challenge, clinical symptom was observed in none of the piglets in PRV B-gD&gC^S^ group. This result indicated that PRV B-gD&gC^S^ was safe and provided effective protection against virulent PRV variant. Vaccine has played an important role to eradicate PRV, and it is crucial to reduce or prevent virus shedding post infection with virulent strain. Virus shedding is one of the important parameters to evaluate the efficacy of PRV vaccine [29]. The efficient reduction of virus shedding with virulent strain infection is crucial to achieve PRV eradication. In this study, the piglets of PRV B-gD&gC^S^ group did not excrete PRV post immunization. Two piglets in piglets of PRV B-gD&gC^S^ group did not shed virus post challenge, and the other three pigs recorded lower titers of shed virus compared with pigs in the Bartha group.

Mutations of glycoprotein genes may directly lead to the impaired protection of Bartha-K61 vaccination against PRV variant. In a previous study, the insertion or deletion mutations was found in gB, gC, gD, gE, gI and gN genes of AH02LA strain compared with Bartha [20]. gB, gC and gD of PRV have been suggested as major immunogenic antigen that induce protective immunity against PRV infection [14, 15]. gB of PRV is important for inducing cell-mediated immune responses [16]. gD glycoprotein is involved in inducing the virus-neutralizing antibody, but not for the induction of cell-mediated immune response [16]. PRV gC is an important target antigen specific for cytotoxic T lymphocytes in pig and mice [30]. The combination of high levels of antibody and cell-mediated immunity is more likely to prevent complete virus shedding. The substituted gD&gC of AH02LA may not induce sufficient antibody and cell-mediated immunity for all piglets against AH02LA challenge. Therefore, the genetic divergence in other immunogenic antigen (except gC and gD) of AH02LA and Bartha-K61 may explain that three piglets of PRV B-gD&gC^S^ group had reduced virus shedding, but did not completely stop virus shedding post challenge.

## Conclusion

We constructed an infectious BAC clone of Bartha-K61 and a gD&gC-substituted pseudorabies virus PRV B-gD&gC^S^. PRV B-gD&gC^S^ is safe for piglets and provides complete clinical protection against a pseudorabies variant (AH02LA) challenge. More importantly, it effectively reduces and may even prevent virus shedding in piglets challenged with virulent AH02LA strain. Future studies involving neutralizing antibody and cell-mediated immunity analysis are necessary to elucidate the mechanism underlining the virus shedding reduction in piglets vaccinated with PRV B-gD&gC^S^.

## Methods

### Viruses, cells and plsmids

PRV stain AH02LA was isolated and identified in our lab (CGMCC No. 10891) [19]. The PRV Bartha-K61 strain was isolated from the nasal mucosa of piglets vaccinated with Bartha-K61 strain in 2016, which exhibited 100% homology with the published sequence of gB, gC, gD, gG, gK and gM genes in Bartha-K61 genome (data not shown). Swine testicular (ST) cells and chicken embryo cells (CEC) were cultured in Dulbecco’s Modified Eagle’s Medium (Gibco, USA) supplemented with 10% fetal calf serum (Gibco) and 1% penicillin and streptomycin (Sigma–Aldrich, USA) or medium containing 2% fetal bovine sera (FBS) after transfection or infection at 37 °C with 5% CO_2_ in a humidified incubator.

The left homologous arm (H1^B^) and right homologous arm (H2^B^) of gC were amplified from the Bartha-K61. Both fragments H1^B^ and H2^B^ were cloned into pUC19 using restriction enzyme sites in the primers (Table 2), and recombinant plasmid was named pUC19-H1^B^-H2^B^. To construct transfer vector plasmid pHA2-Puc19- H1^B^-H2^B^, a mini-F vector from plasmid pDS-pHA2 [31] was cloned into the *Pac* I site in pUC19- H1^B^ -H2^B^. The fragments H1-H2-gC^A^ (AH02LA) and gD (AH02LA) were amplified from the AH02LA by PCR and cloned into T Vector pMD19 (Takara), and the resulting constructs were named H1-H2-gC^A^-T and gD^A^-T, respectively. The plasmid gD^A^-KAN-T containing gD^A^ and a kanamycin resistance gene inserted at the *Sac* I restriction site was constructed by cutting and ligating for further *En Passant* recombination (Fig. 1).

**Table 1:** Results of safety and efficacy test

Groups			A	B	C	D
Virus tested			B-gD&gC^S^	Bartha K61	/	/
Dosage			1 × 10^6^ TCID_50_		PBS	/
Inoculation route			Intramuscularly			
ELISA antibodies against PRV gB or gE	B.V.	gB+	0^a^/5^b^	0/5	0/5	0/5
		gE+	0/5	0/5	0/5	0/5
	7d P.V.	gB+	5/5	5/5	0/5	0/5
		gE+	0/5	0/5	0/5	0/5
	14 d P.C.	gB+	5/5	5/5	2/5	0/5
		gE+	5/5	5/5	2/5	0/5
Clinical signs P.V.			0/5	0/5	0/5	0/5
Fever frequency (≥40.5 °C) P.V.			0/5	0/5	0/5	0/5
Virus shedding P.V.			0/5	0/5	/	/
Fever frequency (≥40.5 °C) P.C.			0/5	0/5	5/5	0/5
Clinical signs P.C.	Morbidity		0/5	0/5	5/5	0/5
	Duration (days)		/	/	4~12	/
	Mortality		0/5	0/5	3/5	0/5
Virus shedding P.C.	Frequency		3/5	5/5	5/5	0/5
	Titer (TCID_50_/g)		10^2.49^~10^6.19^	10^1.30^~10^9.82^	10^1.30^~10^8.83^	/
	Duration (days)		1~8	4~10	3~10	/
Lung lesions P.C.			0/5	0/5	5/5	0/5

*B.V.* before vaccination, *P.V.* post vaccination, *P.C.* post challenge, *gB+* antibodies against PRV gB positive, *gE*+ antibodies against PRV gE positive, “/”, not applicable; ^a^the number of piglets positive; ^b^the number of piglets in the group

**Table 2:** Primers for PCR or sequencing

Primer	Sequence (5′- 3′)
Bartha-gC-H1-F	CATGAATTCGCGTCGACGATGCTGCTCGG
Bartha-gC-H1-R	TCAGGTACCGTTAATTAACAAAAACGACGCGAGCGTGGG
Bartha-gC-H2-F	CGAGGATCCGTTAATTAACCACGTCGAATCAATAAACG
Bartha-gC-H2-R	CACAAGCTTCCGTGAACAACATGCTGCTG
AH02LA-gD-F	TCGATCTACATCTGCGTCGC
AH02LA-gD-R	ATCATCGACGCCGGTACTGC
Kan ins gD F	TCCGTCGACGGCGTGAACATCCTCACCGACTTCATGGTGGCGCTCCCCGGGATGACGACGATAAGTAGGGATAAC
Kan ins gD R	TACGTCGACGGGTAATGCCAGTGTTACAACCA
Bartha ins gD^A^-kan F	CATGCTGCTCGCAGCGCTAT
Bartha ins gD^A^-kan R	GCCGGTACTGCGGAGGCTAC
AH02LA gC flanking F	GCGTCGACGATGCTGCTCGG
AH02LA gC flanking R	CCGTGAACAACATGCTGCTG
SEQ-AH02LA gC F	ACTTTTTCACGACGCCGCGG
SEQ-AH02LA gC R	AAGCGGACCTCGAAGGTCTC

### DNA extraction and transfection

As described earlier, viral DNA was purified from infected CEC or ST cells by SDS–proteinase K extraction [32]. Transfection of virus, plasmid or BAC DNA was performed using Lipofectamine 2000 reagent (Invitrogen) according to manufacturer’s instructions.

### Multi-step growth kinetics

Multi-step growth kinetics of virus was tested on ST cells with a multiplicity of infection (MOI) of 0.01 as described previously [32]. The culture cells were collected at different time points and serial dilutions were propagated in cell monolayers. Virus titers were calculated as 50% tissue culture infectious dose (TCID_50_). Growth kinetics curve was performed in triplicate.

### Bacterial manipulations

Commercial chemical competent *E.coli* cells DH5α (Takara), Electrocompetent MegaX DH10B T1^R^ Electrocomp cells (Invitrogen) and GS1783 (provided by professor Nikolaus Osterrieder) were used for bacterial manipulations. Electroporation was carried to transform viral or BAC DNA into *E.coli* cells as described earlier [31, 33, 34].

### PCR and sequencing

Bartha-gC-H1-F/Bartha-gC-H1-R and Bartha-gC-H2-F/Bartha-gC-H2-R (Table 2) were designed to amplify the homologous sequences upstream and downstream of gC with Bartha-K61 strain DNA as template. Primers AH02LA-gD-F/AH02LA-gD-R (Table 2) for gD of PRV AH02LA strain were used to carry out the PCR. To insert a kanamycin resistance gene into plasmid gD^A^ -T, a pair of specific primers (Kan ins gD F and Kan ins gD R; Table 2) were designed with two Sac I restriction sites that were added in both terminals for cutting and ligation. Bartha ins gD^A^-kan F and Bartha ins gD^A^-kan R (Table 2) were used to insert gD^A^-kan into the Bartha BAC clone through the *En Passant* protocol. A pair of primers (AH02LA gC flanking F and AH02LA gC flanking R; Table 2) were used to amplify a fragment from the AH02LA that included the gC gene and two homologous flanking sequences of gC for gC substitution (~ 1 kpb). Nucleotide sequencing reactions were performed by Tsingke Biotech. (Nanjing, China).

### Generation of a PRV Bartha-k61 infectious clone

A Bartha-K61 infectious clone was generated following the general technique of BAC [35]. Briefly, PRV Bartha-K61 DNA and pHA2-Puc19-H1^B^-H2^B^ was co-transfected into ST cells, ensuring mini-F sequences insertion in the lieu of gC. After green plaques were observed under UV light (488 nm), plaque purification was carried out to obtain the homogeneous viruses, named B-mini-F. DNA of B-mini-F was transferred into *E.coli* DH10B competent cells (Invitrogen) by electroporation. Restriction fragment length polymorphism (RFLP) with *Hind* III and *Sph* I were determined to select positive clone of pB-mini-F, which was then electroporated into *E.coli* GS1783 for mutagenesis of Bartha-K61 genome [35].

### Construction of gD&gC-substituted pseudorabies virus (PRV B-gD&gC^S^)

To replace the gD of Bartha-K61, gD^A^-KAN was insert into gD area of pB-mini-F through the *En Passant* [33]. Briefly, PCR was performed with a pair of primers (AH02LA ins gD-KAN F and AH02LA ins gD-KAN R; Table 2) to amplify gD^A^-KAN, which contains 40-bp homologous sequences of Bartha-K61. PCR product was digested with Dpn I and was electroporated into GS1783 with pB-mini-F to achieve the first recombination at the gD sites. Through the second recombination, the target recombinant pB-gD^S^-mini-F clone was selected with the kanamycin resistance gene deletion (Fig. 1). Selected clones were confirmed by RFLP with *Hind* III and *Sph* I. The replaced gD gene was identified through PCR and sequencing. To obtain the gD&gC-substituted pseudorabies virus (PRV B-gD&gC^S^), ST cells were transfected with pB-gD^S^-mini-F DNA and H1-H2-gC^A^ (generated as described above). One to two days after transfection, non-fluorescent plaques (488 nm) were selected and purified to obtain a homogeneous population. The substituted gD and gC genes were confirmed using PCR and sequencing.

### Safety and immunogenicity of PRV B-gD&gC^S^ in piglets

This animal study was approved by the Institutional Animal Care and Use Committee at the Jiangsu Academy of Agriculture Sciences (authorization number SYXK (Su) 2015–0019) and Zhengzhuquan Pig Breeding Farm, and performed in strict accordance with the guidelines provided by the Institutional Biosafety Committee. Twenty 28 days piglets free of PRV, porcine reproductive and respiratory syndrome virus, porcine parvovirus, porcine circovirus 2 and classical swine fever virus were obtained from Zhengzhuquan Pig Breeding Farm, Nanjing, China. All piglets were randomly assigned to four groups (*n* = 5 each) based on equal body weights, and housed in separated facilities. Piglets were inoculated intramuscularly with 2 mL of PRV B-gD&gC^S^ (10^6.0^ TCID_50_/dose, group A), Bartha-K61 (10^6.0^ TCID_50_/dose, group B), PBS (group C) or PBS (group D). One week after inoculation, groups A, B and C were challenged intranasally with 2 LD_50_ PRV AH02LA to assess protection. All piglets were monitored daily for body temperature, clinical signs, and virus shedding from1 day post inoculation (dpi) to 14 day post challenge (dpc). During the experimental period, the temperature of the pig house was maintained at 28 ± 2 °C. All piglets were fed twice daily (at 8:00 and 17:00) and had free access to water via a low-pressure nipple drinker. Upon completion of the experiments, all animals were euthanized by intravenous injection of pentobarbital sodium (100 mg/kg).

### Serological tests for gB and gE antibodies

Blood samples of all piglets were collected before vaccination, at 7 dpi and at 14 dpc, respectively. Serum was obtained by centrifuging at 2000 rpm for 10 min at 4 °C. PRV specific gE or gB antibody detection ELISA kit (IDEXX, Maine, USA) were used to detect gE or gB antibody in the serum according to the manufacturer’s instructions.

### Detection of virus titers in the nasal swab

Nasal swab samples of all piglets were collected and weighed daily from 0 dpi to 14 dpc. After shaking (2 min at 8, 000 rpm) and freeze-thaw cycles (− 70 °C and 37 °C), samples were centrifuged (15 min at 10, 000 rpm) and ten-fold serial dilutions of supernatants were used to infect ST cells to determine the viral titers.

## Abbreviations

BAC: Bacterial artificial chromosome
CEC: Chicken embryo cell
dpc: Day post challenge
dpi: Day post inoculation
*E.coli*: *Escherichia coli*
ELISA: Enzyme-linked immunosorbent assay
gB: Glycoprotein B
gC: Glycoprotein C
gD: Glycoprotein D
LD50: 50% lethal dose
MOI: Multiplicity of infection
PCR: Polymerase chain reaction
PRV: Pseudorabies virus
RFLP: Restriction fragment length polymorphism
ST cells: Swine testicular cells
TCID_50_: 50% tissue culture infectious dose
